# Famous Landmark Identification in Amnestic Mild Cognitive Impairment and Alzheimer's Disease

**DOI:** 10.1371/journal.pone.0105623

**Published:** 2014-08-21

**Authors:** Katerina Sheardova, Jan Laczó, Martin Vyhnalek, Ross Andel, Ivana Mokrisova, Kamil Vlcek, Jana Amlerova, Jakub Hort

**Affiliations:** 1 International Clinical Research Center, St. Anne's University Hospital Brno, Brno, Czech Republic; 2 Memory Clinic, Department of Neurology, Charles University in Prague, 2nd Faculty of Medicine and University Hospital Motol, Prague, Czech Republic; 3 School of Aging Studies, University of South Florida, Tampa, Florida, United States of America; 4 Department of Neurophysiology of Memory, Institute of Physiology, Academy of Sciences of the Czech Republic, Prague, Czech Republic; University of Lancaster, United Kingdom

## Abstract

**Background:**

Identification of famous landmarks (FLI), famous faces (FFI) and recognition of facial emotions (FER) is affected early in the course of Alzheimer's disease (AD). FFI, FER and FLI may represent domain specific tasks relying on activation of distinct regions of the medial temporal lobe, which are affected successively during the course of AD. However, the data on FFI and FER in MCI are controversial and FLI domain remains almost unexplored.

**Objectives:**

To determine whether and how are these three specific domains impaired in head to head comparison of patients with amnestic MCI (aMCI) single domain (SD-aMCI) and multiple domain (MD-aMCI). We propose that FLI might be most reliable in differentiating SD-aMCI, which is considered to be an earlier stage of AD pathology spread out, from the controls.

**Patients and Methods:**

A total of 114 patients, 13 with single domain (SD–aMCI) and 30 with multiple domains (MD–aMCI), 29 with mild AD and 42 controls underwent standard neurological and neuropsychological evaluations as well as tests of FLI, FER and FFI.

**Results:**

Compared to the control group, AD subjects performed worse on FFI (p = 0.020), FER (p<0.001) and FLI (p<0.001), MD-aMCI group had significantly worse scores only on FLI (p = 0.002) and approached statistical significance on FER (0.053). SD-aMCI group performed significantly worse only on FLI (p = 0.028) compared to controls.

**Conclusions:**

Patients with SD-aMCI had an isolated impairment restricted to FLI, while patients with MD–aMCI showed impairment in FLI as well as in FER. Patients with mild dementia due to AD have more extensive impairment of higher visual perception. The results suggest that FLI testing may contribute to identification of patients at risk of AD. We hypothesize that clinical examination of all three domains might reflect the spread of the disease from transentorhinal cortex, over amygdala to fusiform gyrus.

## Introduction

Alzheimer disease (AD) is considered to be a continuum from preclinical stage through the prodromal stage represented by mild cognitive impairment (MCI) syndrome to the dementia syndrome [1], [2], [3]. The difference between MCI and dementia is in preserved functional capacity of MCI individuals whereas cognitive impairment is present in both stages. It is well accepted that beside the impairment of episodic memory, there are also other cognitive domains affected in early stages of AD, such as semantic memory, executive functions, attention, language, visuo-constructive skills and spatial navigation [4], [5], [6], [7].

The individuals with MCI form a heterogeneous group, where those with memory impairment – amnestic MCI (aMCI), seem to be more vulnerable to convert to AD with estimated average rate of conversion 12% per year [8]. Some of aMCI subjects present with isolated memory impairment – aMCI single domain (SD-aMCI), while others present with impairment in additional domains to memory – aMCI multiple domain (MD-aMCI) [9]. Individuals with MD-aMCI are more likely to convert to dementia than SD-aMCI subjects [10] and might thus represent a more advanced stage of AD pathology than SD-aMCI subjects. However, not all of the individuals with aMCI syndrome convert to dementia; some may remain stable or even reverse back to normal cognition. Therefore much effort is spent to identify subjects at higher risk with putative underlying AD pathology who are considered to be at prodromal stages of AD.

Besides the structural and functional neuroimaging, focused on the hippocampus and related structures, and the cerebrospinal fluid assessment of amyloid-β peptide, tau, and phosphorylated tau proteins, specific memory tests play an important role in identification of the high risk MCI subjects. Specifically, “amnestic syndrome of the hippocampal type” [11] seems to be characteristic for prodromal stages of AD [12], [13]. Besides clinically well-established episodic memory tests [14], there has been ongoing search for novel instruments aiming even for earlier AD related changes with highest possible sensitivity and specificity.

Higher visual perception, which includes identification and recognition of faces and landmarks as well as recognition of facial emotions, is dependent on the medial temporal lobe structures that are affected early in the course of AD. There is some empirical evidence that these domains might be affected already in the MCI subjects [15], [16], [17].

Studies on famous faces identification (FFI) report consistently impairment of this domain in subjects with dementia due to AD [18], [19], [20] while studies with MCI subjects report rather inhomogeneous results [15], [16], [21], [22].

Another domain affected early in patients with AD is recognition of facial emotions (FER) [17], [23]. Reports on FER impairment in MCI are controversial [24], [25], [26], [27]. However, evidence favors the hypothesis that worse FER is associated with MCI compared to normal aging [28].

Only very sporadic data exists on famous landmark identification (FLI) in AD – casuistic report is available of an AD patient with impaired discrimination between famous and unknown buildings despite of preserved identification of faces [29]. The single study with FLI in MCI [16] found that MCI subjects were impaired in naming of famous buildings, famous faces, and of well-known objects compared to controls.

The inconsistent results of FLI, FFI and FER impairment in MCI might be the result of different study populations: Some studies compared subgroups of patients with amnestic MCI while the others also included those with non-amnestic MCI. In addition, these studies use different paradigms exploring each specific domain. Some studies rely on testing the naming of famous faces/objects which also involves some semantic processing [15], [16] while others use face matching tasks, comparing similarities or differences in facial features or emotions [17], [21], [22].

Recognizing famous faces, famous landmarks and emotions is probably domain specific task. Imaging studies in cognitively healthy subjects have shown category specific activation in medial temporal structures during tasks with buildings, emotion and famous faces recognition. Parahippocampal/lingual gyri are more responsive to buildings [30]; amygdala and adjacent cortex are activated during emotion recognition [31], [32], while the fusiform gyri are preferentially responsive to famous faces [22], [33].

Clinical staging of AD corresponds with spread of tau pathology (formation of typical argyrophilic neurofibrillary tangles and neuropil threads within the neurons) characterized in Braak staging [34], where stage I-IV corresponds with the spread of pathology in the direction from transentorhinal and parahippocampal cortices, to hippocampus, fusiform gyrus and beyond [35]. We suggest that the impairment in identification of these domain specific categories (FER, FFI and FLI) could appear based on their structural correlates in a timely manner during the course of AD following the Braak stages. We have used well defined groups of patients (SD-aMCI, MD-aMCI and mild AD).

The aim of our study was to perform head to head comparison of these three domain specific paradigms relying on various medial temporal lobe structures in well-defined subgroups of aMCI and mild AD and to assess whether these tests can reliably distinguish SD-aMCI and MD-aMCI from controls. Based on the domain specific structural correlates, we expected that all 3 tasks will be affected in mild AD, while only FER and FLI would be impaired in aMCI compared to controls. Assuming that SD-aMCI might be an earlier stage of AD pathology then MD-aMCI, we hypothesize that FLI, which is relying on the parahippocampal gyrus, a brain region affected very early in the course of AD, might be more reliable in distinguishing SD-aMCI from controls.

## Materials and Methods

### 1. Participants

The study was approved by the institutional ethics committee of University Hospital Motol and all participants provided a written informed consent. In demented people a research consent form was approved and signed on the patient's behalf by the caregiver. A total of 114 subjects were recruited at the Memory Clinic of the University Hospital Motol, 29 patients with mild AD, 43 patients with aMCI (13 SD–aMCI and 30 MD–aMCI), and 42 cognitively healthy controls. Cognitively healthy participants were recruited from the older adults attending University of the Third Age at Charles University in Prague or from relatives of patients of the Memory Clinic, Motol University Hospital in Prague. Subjects with memory complaints, history of neurological or psychiatric disease, psychiatric medication usage, or abnormal neurological examination including gait or movement difficulties were not included. Participants meeting DSM IV-TR criteria for dementia, Petersen's criteria for MCI [36] or scoring more than 1.5 SD below the age- and education-adjusted norms on neuropsychological examination were not included into the control group. MCI and AD subjects were referred to the clinic by general practitioners, neurologists, psychiatrists, and geriatricians. AD patients met the NINDS ADRDA diagnostic criteria and all participants with aMCI met published revised clinical criteria for MCI [36] including memory problem reported by patient or caregiver, generally intact activities of daily living, evidence of cognitive dysfunction with predominant memory involvement on neuropsychological testing, and absence of dementia. The aMCI patients scored in memory tests 1.5 standard deviation points below the mean of age- and education-adjusted norms. The aMCI subjects were further classified into SD-aMCI and MD-aMCI. SD-aMCI patients had an isolated memory deficit. Cognitive impairment in attention and executive function, language skills, or visuospatial skills in addition to memory impairment was used to classify subjects as having MD-aMCI. Patients with a Hachinski Ischemic Scale score >4 [37] or with a history of other neurological or psychiatric disorders including depression – scoring >5 in the short 15 items Geriatric depression scale [38] were not included in the study. All participants underwent standard neurological and laboratory evaluations, 1.5T magnetic resonance brain imaging, clinical scaling Mini Mental State Examination (MMSE) [39] and complex neuropsychological testing. Patients with extensive vascular changes – Fazekas score 3 [40], lacunar stroke, meningioma or other severe structural pathology on brain MRI were excluded from the study.

### 2. Neuropsychological evaluation

The neuropsychological battery was covering 1) memory, measured by Auditory Verbal Learning Test trials 1–6 and the Auditory Verbal Learning Test Delayed Recall [41], [42], Rey-Osterrieth Complex Figure Recall condition [43] and modified version of FCSRT called Enhanced Cued Recall (ECR test in Czech validated version) [13], [44]; 2) attention/processing speed, measured with the Digit Span Backwards [45] and Trail Making Test A [46]; 3) executive functions, measured with the Trail Making Test B [46] and Controlled Oral Word Association (COWAT) test [47]; 4) language, measured with the Boston Naming Test [48]; and 5) visuospatial functions measured with the Rey-Osterrieth Complex Figure Copy condition [43]. The score for each domain was expressed as a unit weighted composite score from the relevant tests. The Trail Making Test subtasks, which are expressed in seconds to completion, were reverse scored before the means were generated. Boston Naming Test scores were used only for MCI patient classification. The MMSE was administered to measure global cognitive functions.

### 3. Test of famous faces identification

This test was adapted from Keane's study [49] and adjusted for a Czech population [50]. Faces of 10 highly famous persons (politicians, actors, musicians, etc.) and 10 unfamiliar faces were presented to the subjects in a fixed pseudo-random order. We used pictures of famous people from visual media. For each face, the participant decided whether the person was familiar or not. The performance was measured by the number of faces correctly recognized as familiar or unfamiliar (correct rejections) with possible scores ranging from 0–20. The battery of famous faces was composed only from Czech personalities. The test was administered by a single qualified test administrator to avoid interrater variability.

### 4. Test of famous landmarks identification (Fig.1)

The famous objects were depicted considering Czech generally well known buildings and international buildings well-known within the Czech population. Identification of these objects was previously tested on a set of elderly cognitively healthy volunteers. Items which were not recognized by 20% or more of the volunteers were not included in the test. The administration of the test was fully computer based to avoid interrater variability.

**Figure 1:**
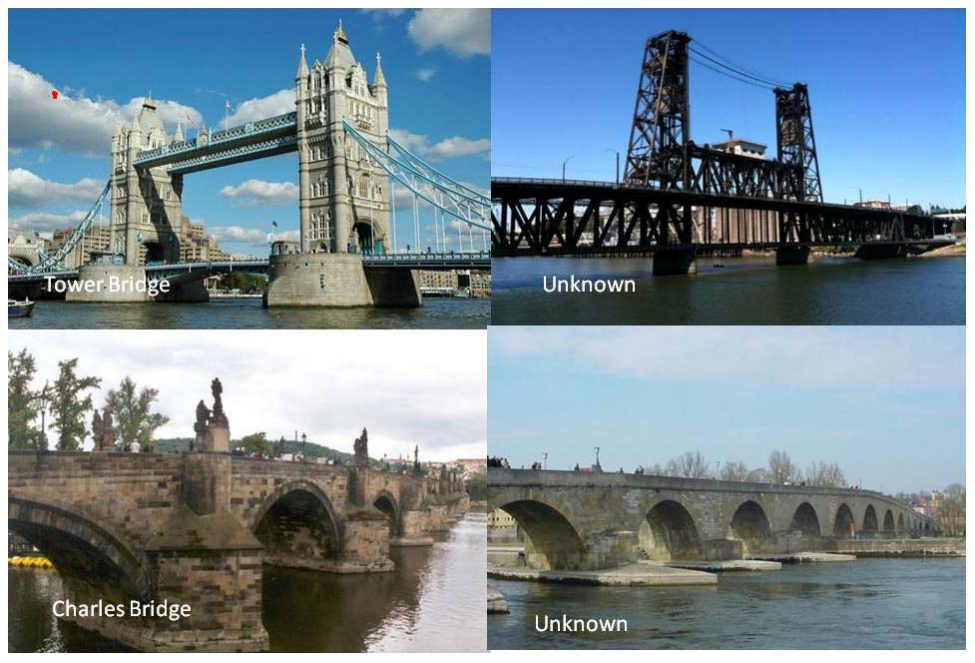
Test of famous landmarks identification. Illustration of two famous places for the Czech population and two similar but unfamiliar places. For each place, the participant decided whether the place was familiar or not.

Pictures of 25 highly famous places worldwide (buildings, bridges, statues etc.) and 25 matched pictures of unfamiliar places were presented in a fixed pseudo-random order. For each place, the participant decided whether the place was generally familiar or not. Each correctly recognized place as familiar or unfamiliar (correct rejections) was scored with one point – score range 0-50.

### 5. Test of facial emotions recognition

Pictures from the Ekman and Friesen series [51] representing five basic emotions, i.e., happiness, anger, sadness, fear and disgust were used to measure recognition of facial emotions. Each category of the five emotions was presented by using five pictures of different faces. The description of each emotion was printed under each picture in a random order in multiple choices. The participants were asked to point to the emotion which correlated best with the facial expression shown above. There were 25 trials (five for each emotion) with possible scores ranging from 0–25. The emotions were randomly presented and no target picture was used more than once.

### 6. Statistical evaluation

Inferential statistics involved a one-way analysis of variance (ANOVA) to evaluate between-group differences in age, MMSE, and neuropsychological tests. The χ^2^ test was used to evaluate differences in proportions (gender). The between-group differences in the main analyses with FFI, FER and FLI were evaluated using a general linear model (GLM). As the groups differed in the level of education, education was used as a covariate in these models. In the second GLM model we controlled for global cognitive functioning by adding a MMSE score to the previous model. All post hoc analyses were carried out with the Sidak test.

In the correlation analyses, first, zero-order Pearson correlation with Holm-Bonferroni correction for multiple comparisons was used to assess the relationship between the FFI, FER and FLI and neuropsychological tests. Subsequently, partial Pearson correlation with Holm-Bonferroni correction was used to control for the effect of group membership. Due to low variability of the scores across the groups, we used all participants within one correlation analysis. This step did not affect the results. The significance level was set at two-tailed 0.05. All analyses were run using SPSS 13.0 for Windows.

## Results

The groups did not differ in age (F[3,110] = 2.11; p = 0.103) and gender (χ^2^(3) = 3.03; p = 0.387), but in education (F[3,110] = 8.65; p<0.001), specifically AD (p<0.001) and SD-aMCI (p = 0.023) had less years of education than the control group. The demographical and neuropsychological characteristics are presented in Table 1.

**Table 1:** Demographic characteristics of the groups.

	Controls (n = 42)	SD-aMCI (n = 13)	MD-aMCI (n = 30)	mild AD (n = 29)	P value	Effect size
Age	71.55 (4.95)	72.62 (7.68)	71.93 (9.18)	74.41 (8.44)	0.103^a^	0.054^c^
Sex W/M	25/17 (0.60)	9/4 (0.69)	13/17 (0.43)	17/12 (0.59)	0.387^b^	0.162^d^
Education	15.79 (2.59)	13.23 (2.89)*	14.83 (3.44)	12.59 (2.21)***	<0.001^a^	0.190^c^
MMSE	28.54 (1.44)	27.04 (2.32)	26.02 (2.86)***	19.79 (3.26)***	<0.001^a^	0.617^c^
FCSRT	15.88 (0.33)	12.25 (2.71)	13.81 (3.03)*	9.00 (1.41)***	<0.001^a^	0.362^c^
AVLT 1-6	58.41 (12.15)	30.75 (9.71)***	29.00 (6.57)***	30.0 (2.83) ***	<0.001^a^	0.701^c^
AVLT 30	10.18 (3.38)	1.25 (1.49)***	2.24 (1.64)***	0.50 (0.71) ***	<0.001^a^	0.752^c^
ROCF - R	18.38 (6.17)	6.80 (4.10)***	8.95 (5.16)***	1.50 (2.12)***	<0.001^a^	0.501^c^
DSB	4.94 (0.97)	4.50 (1.41)	4.19 (1.66)	4.50 (0.71)**	0.003^a^	0.193^c^
TMT A	40.68 (8.72)	45.63 (30.66)	60.14 (23.80)	65.00 (32.53)**	0.001^a^	0.172^c^
TMT B	87.56 (19.74)	113.75 (36.51)	186.62 (119.79)**	355.00 (205.06)***	<0.001^a^	0.353^c^
COWAT	43.24 (11.86)	37.88 (9.99)	30.76 (10.40)**	25.50 (7.78)***	<0.001^a^	0.249^c^
ROCF - C	31.76 (1.79)	31.88 (2.03)	26.95 (5.24)*	16.75 (9.55)***	<0.001^a^	0.448^c^
BNT err.	2.50 (1.89)	5.25 (2.44)	6.19 (3.81)*	12.40 (5.76)***	<0.001^a^	0.800^c^
FFI	18.61 (1.48)	18.38 (1.66)	17.66 (2.72)	16.79 (2.90)*	0.017	0.098^c^
FER	21.93 (2.23)	20.00 (2.20)	20.03 (2.54)	17.13 (4.02)***	<0.001	0.223^c^
FLI	42.27 (3.79)	37.62 (4.25)*	37.90 (4.72)**	33.17 (5.91)***	<0.001	0.317^c^

Mean values (SD); Auditory Verbal Learning Test (AVLT) trials 1–6 and AVLT Delayed Recall (AVLT 30), Rey-Osterrieth Complex Figure Copy (ROCF - C) and Recall (ROCF – R), Free and Cued Selective Reminding Test (FCSRT) total recall, Digit Span Backward (DSB), Trail Making Test (TMT) A and B, Controlled Oral Word Association (COWAT), Boston Naming Test errors (BNT err.); one-way ANOVA - between-group differences.

a ANOVA, ^b^X^2^ test, ^c^Partial eta ^2^, ^d^Cramér's V, * p<.05, **<.01, ***<.001 (compared to the control group) Note: Partial eta^2^ of 0.2 corresponds to Cohen's d of 1.0 with our sample size, Cramér's V of about 0.175 corresponds to Cohen's d of 0.356.

There was a moderate positive correlation between FER and FLI, and a low positive correlation between FFI and FLI and between FFI and FER. Correlations between FFI, FER, FLI, MMSE and cognitive domains are presented in Table 2. When we controlled for a group membership in the correlation analyses, only a low positive correlation between FER and FFI and between FER and FLI together with a moderate positive correlation between FLI and MMSE remained significant; see Table 2.

**Table 2:** Correlations of FFI, FER and FLI with cognitive domains (EGM – correlations controlled for effect of group membership).

		FFI	FER	FLI
MMSE	EGM	0.127	0.114	0.407**
		0.313*	0.411**	0.681***
memory	EGM	0.220	0.171	0.139
		0.370**	0.438**	0.531***
attention	EGM	0.248	0.248	0.177
		0.309*	0.333*	0.299*
executive	EGM	0.092	0.228	0.245
		0.247	0.425**	0.511***
visuospatial	EGM	−0.094	−0.110	0.228
		0.104	0.181	0.504***

* p<0.05, **<0.01, ***<0.001 values in bold indicate significant correlations after Holm-Bonferroni correction for multiple comparisons. The tests used for testing each cognitive domain are closely described in the methods.

**Figure 2:**
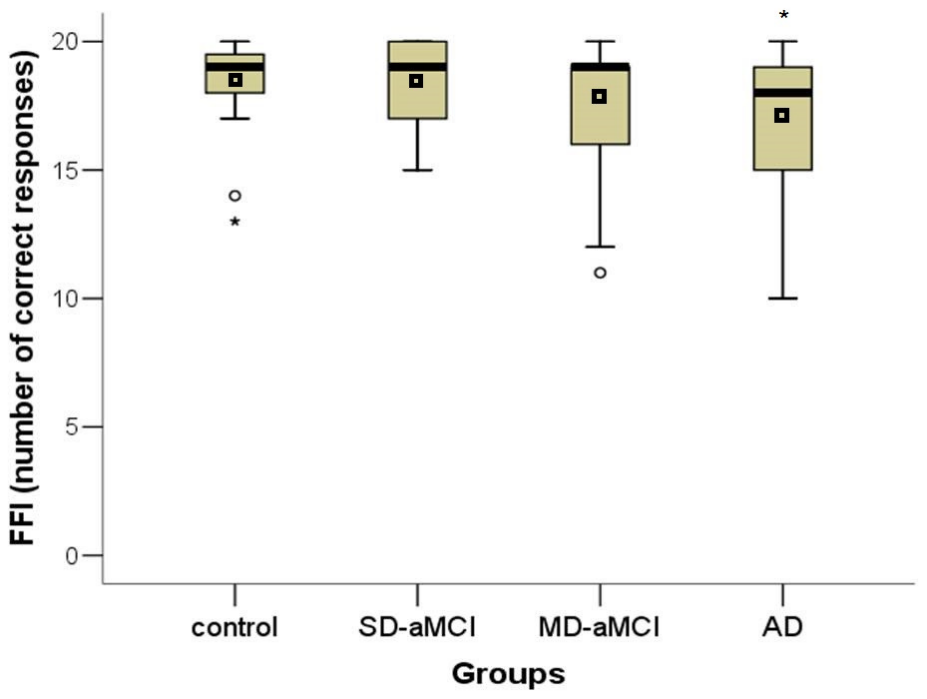
Differences across groups in the FFI test. The total number of faces correctly recognized as familiar or unfamiliar (correct rejections) in each group is depicted. * p<0.05. Note: mean, median and interquartile ranges characterise performance of each group. FFI  =  Test of famous faces identification, SD-aMCI  =  single domain amnestic mild cognitive impairment, MD-aMCI  =  multiple domain amnestic mild cognitive impairment, AD  =  Alzheimer's disease dementia.

**Figure 3:**
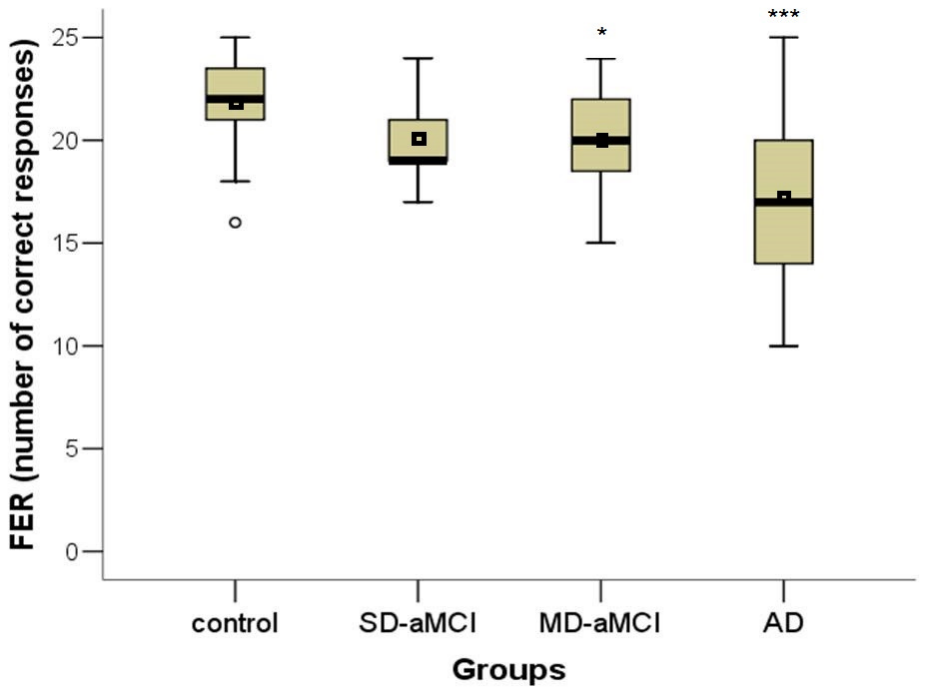
Differences across groups in the FER test. The total number of correctly recognized emotions in each group is depicted. * p<0.05, *** p<0.001. Note: mean, median and interquartile ranges characterise performance of each group. FER  =  Test of facial emotions recognition, SD-aMCI  =  single domain amnestic mild cognitive impairment, MD-aMCI  =  multiple domain amnestic mild cognitive impairment, AD  =  Alzheimer's disease dementia.

In the main GLM analysis controlling for education, we found significant main effects for group in FFI (F[3,109] = 3.54; p = 0.017), FER (F[3,109] = 12.00; p<0.001) and FLI (F[3,109] = 15.60; p<0.001) tests. Specifically, the SD-aMCI was impaired only in FLI (p = 0.028) compared to the control group. Further, the MD-aMCI had lower performance in FLI (p = 0.002) compared to the control group. Differences between the MD-aMCI and the control group in FER approached statistical significance (p = 0.053). Finally, the AD group had lower performance in all three main tests, FFI (p = 0.020), FER (p<0.001) and FLI (p<0.001), compared to the control group. There were no differences between the SD-aMCI and MD-aMCI groups. For the differences in the performance among the groups see in Figure 2, 3, 4. In the second GLM analysis controlling for education and MMSE score, the main significant effect remained for the FLI (F[3,108] = 5.97; p = 0.001) and FER (F[3,108] = 5.38; p = 0.002) tests, but not for the FFI (F[3,108] = 2.21; p = 0.091). Specifically, the differences between the SD-aMCI and the control group approached statistical significance in FLI (p = 0.057). Further, the differences between the MD-aMCI and the control group remained significant for FLI (p = 0.013), but not for FER (p = 0.083). Finally, the differences between the AD and the control group remained significant for FER (p = 0.001) and FLI (p = 0.001) tests. The differences between the SD-aMCI and MD-aMCI groups remained non-significant.

**Figure 4:**
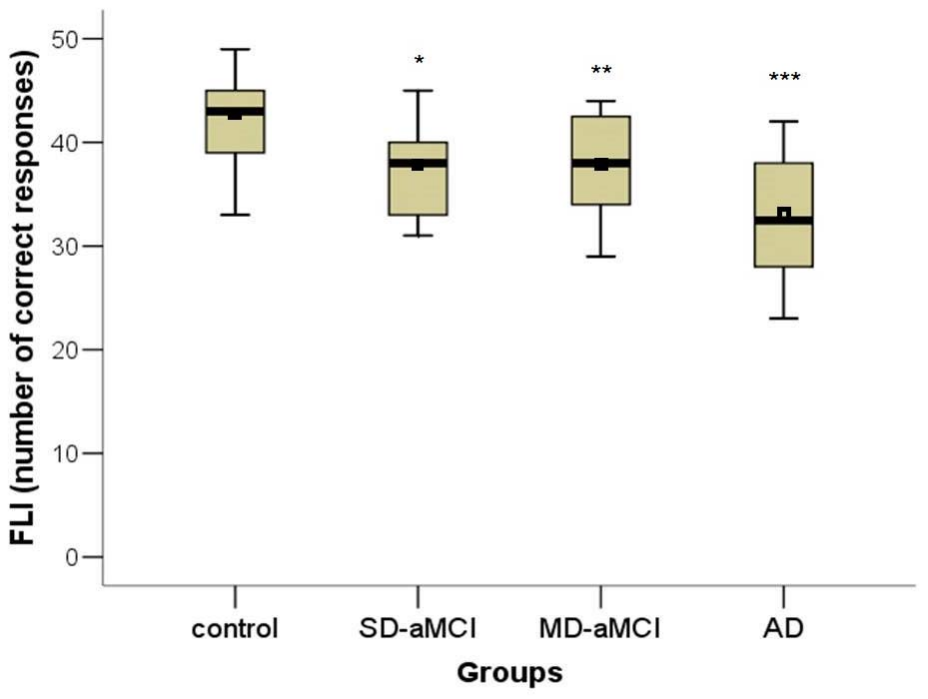
Differences across groups in the FLI test. The total number of correctly recognized places as familiar or unfamiliar (correct rejections) in each group is depicted. * p<0.05, ** p<0.01, *** p<0.001. Note: mean, median and interquartile ranges characterise performance of each group. FLI  =  Test of famous landmarks identification, SD-aMCI  =  single domain amnestic mild cognitive impairment, MD-aMCI  =  multiple domain amnestic mild cognitive impairment, AD  =  Alzheimer's disease dementia.

## Discussion

The findings indicate that SD-aMCI patients performed significantly worse than controls on FLI but not on FER and FFI, MD-aMCI scored worse on FLI and approached statistical significance in FER performance. Further, AD patients exhibited impairment in all 3 visual domains. The findings could not be explained by differences in education but were partially modified by MMSE.

In our previous work we have shown that FER but not FFI may be impaired in MD-aMCI and that neither FER nor FFI is impaired in SD–aMCI [27] which is consistent with the results of this study using different patients' cohort. Similar finding was reported from the study of University of California Los Angeles, which also compared two groups of aMCI subtypes [26]. However, FLI seems to be impaired in both SD-aMCI as well as MD-aMCI group of patients compared to controls and no differences in FLI performance seem to be present between SD-aMCI and MD-aMCI patients. This suggests that FLI could be helpful in combination with other scales in cognitive screening for aMCI in geriatric population.

On the contrary, impairment of FFI does not seem to be very sensitive for MCI. Studies with face matching tasks in MCI subjects suggested no differences in the number of correct answers, but only longer completion time when compared to normal controls [21], [22]. This is consistent with our results where no impairment of FFI compared to controls was found in any of the aMCI subtype and both, SD-aMCI as well as MD-aMCI group, performed similarly when compared with each other.

On the other hand, the Barcelona group [15] reported that slight FFI impairment may be predictive of dementia due to AD developed 2 years later and the Cambridge group did report impairment of FFI in MCI [16]. The different results can be explained by using of different paradigm. Both studies relay the testing of these categories on naming faces and/or buildings, which involves a complex processing network including involvement of stored semantic knowledge about the people or buildings. Psychological studies have suggested that the task of fully identifying and naming a famous person is achieved by a cascade of sequential processing stages [52]: the pre-semantic stage, when recognition of famous faces is impaired only in the visual domain, the semantic stage, when loss of biographical information about known people (person-specific semantics) occurs regardless of the stimulus modality; and the post-semantic lexical retrieval stage, when name retrieval is impaired but semantic information is retrieved correctly. In our study however, subjects did not name the faces/buildings, they were just deciding whether the presented item was famous or not. This is similar to paradigm used in a different Cambridge study [19], which indicated that pure recognition and sense of familiarity can occur independently of accessing semantic information.

Results of our present study show that impairment of FLI is present in aMCI subjects and it can discriminate both aMCI subtypes from controls. There are very few studies on recognizing famous or familiar buildings or landmarks in AD and MCI [16], [29]; the results of these studies correspond with our findings of FLI impairment in AD as well as in MCI and to a more pronounced FLI than FFI impairment in these subjects [53].

According to the literature the FLI, FER and FFI depends on various anatomical structures [17], [21], [30], [31], [32], [54] therefore the differences in the impairment of specific domains among the groups of patients with different severity of cognitive impairment might be caused by distinct neuropathological correlates involved in each paradigm. According to Braak and Braak [35], underlying AD pathology spreads gradually; affecting medio-temporal structures in the typical order and clinical staging corresponds with tau pathology and Braak staging [34]. Our results could be interpreted in this context. FLI refers to parahippocampal/lingual gyri [30]. Lesion of the parahippocampal gyrus may lead to inability to recognize salient environmental landmarks during spatial navigation and may thus cause significant spatial navigation deficits [54]. Transentorhinal cortex, a part of parahippocampal gyrus is the first affected by the AD pathology. This corresponds with a view that SD-aMCI is an earlier stage than MD-aMCI, where besides FLI also FER is impaired. FER depends on the function of the amygdala [31], [32] which is affected later in the course of AD [35].

Spreading of the pathology beyond the mesiotemporal structures in subjects with dementia would correspond to our observation that FFI impairment relying on more lateral regions within temporal neocortex [17], [21] was present together with FLI and FER impairment only in demented subjects.

Our study shares limitation with similar studies in the field which is the absence of neuroimaging correlates. Further, we used a relatively small sample size, which could also influence the results. Especially, due to the small sample size we failed to find differences between SD-aMCI and MD-aMCI groups in FER, although MD-aMCI patients seem to be impaired unlike SD-aMCI patients when compared to the control group. We could not exclude problems with familiarity assessment as an influencing factor, similarly like the other studies on familiarity cited in this article. We acknowledge that some studies in aMCI reported difficulties with assessing familiarity in these subjects [55] and over-reliance on familiarity as well [56]. However other studies did not find impaired familiarity-based recognition in contrary to impaired recognition based on recollection in MCI subjects, suggesting that recollection and familiarity might be independent processes associated with distinct anatomical substrates [57], [58]. PET studies also show that the distinction of famous and non–famous stimuli independently of its category [30], [59], [60], [61] relies on anterior temporal pole, which as a part of associative neocortex is affected later in the course of AD pathology spread out (Braak IV). This might suggest that the statistical differences observed in aMCI subjects reflect the domain specific differences in the task rather than difficulties in familiarity assessment. We cannot also exclude a ceiling effect in the FFI task, which could cover up some of the group differences in performance within this task. The selection of participants is limited because the diagnosis of aMCI was based only on a complex neuropsychological examination and no imaging or biochemical biomarkers were used. Therefore we could not exclude subjects which would not convert to AD in a short time.

However, this study has potential implications for future research. We have introduced a new paradigm on famous landmark identification which allows direct comparison with analogical paradigm described in Keane's study [49] on identification of famous faces. This is to our knowledge the first head to head comparison of these 3 paradigms, which allows interpretation of the usefulness of each paradigm for distinguishing aMCI patients from the controls. The tasks of FLI, FER and FFI probably involve segregated neurocognitive networks part of which are affected in prodromal stages of AD and future research is needed to test this hypothesis. Especially studies with the employment of functional neuroimaging would be of a great advantage. The early spread-out of pathology through the visual ventral stream is a specific feature for AD therefore assessment of these domains could also help in early differential diagnosis of AD versus other forms of dementia such as frontotemporal lobar degeneration where ventral visual stream is spared and diffuse Lewy body disease where dorsal visual stream is early involved.

Another important future implication for research would be to assess how FLI impairment correlates with real spatial navigation difficulties. Spatial orientation difficulties is a well-known and stressful feature reported by caregivers of individuals with dementia due to AD and impairment in spatial navigation is one of the early markers of MCI due to AD pathology while it correlates with hippocampal type of memory impairment [62] and with right hippocampal volume [63]. FLI is related to the ability of recognizing landmarks important for navigation. Recent findings indicated that learning and subsequent recalling or recognition of landmarks or famous places may not be dependent on the way how and in which environment they were perceived. In the study addressing this issue [64] similar results were found when landmarks or places visited by subjects were learned in the real-world and virtual environment, respectively, and also when they were subsequently recalled or recognized from photographs and video clips. The more unique an object is within an environment and the more it is perceived as having a stable spatial position, the more likely it is that it will be used as a landmark. Objects rated as more stable (larger and less “portable”) automatically evoked landmark-based neural processes in the study subjects [65]. In line with this, it has also been shown that making spatial judgments with reference to stable environmental objects (e.g., a large buildings) compared with unstable objects (e.g., a ball) elicit greater activity in navigationally relevant medial parietal and temporal brain regions, including the hippocampus (for review see [66], [67]). Objects included in our FLI test fulfil both of these criteria (shape uniqueness and stability) hence could be relevant for testing one part of complex spatial navigation behaviour used in. Objects used for navigation in the neighbourhood and town are usually landmarks learned long time ago. Therefore difficulties in recognizing them as familiar could be part of the problem everyday navigation scenario of AD subjects. Establishing the relationship between FLI and spatial navigation impairment might confirm the usefulness of FLI in assessment in MCI at high risk for conversion to AD dementia. The practical implication may be that being impaired in the FLI can reflect the difficulties with orientation in the real environment, which may contribute to driving impairments and getting lost.

## Conclusions

Our results suggest that the tasks with recognizing famous landmarks, facial emotions and familiar faces involve segregated neurocognitive networks and might be impaired in a time order in relation to the course of AD. Since these tests refer to different brain structures which are considered to be related to various stages of the disease, assessment of FLI, FER and FFI may provide valuable clinical information indirectly reflecting underlying pathology. Future research is needed to match pathological changes, test performance and longitudinal data.
